# A Human Hepatocyte-Bearing Mouse: An Animal Model to Predict Drug Metabolism and Effectiveness in Humans

**DOI:** 10.1155/2009/476217

**Published:** 2009-10-26

**Authors:** Katsutoshi Yoshizato, Chise Tateno

**Affiliations:** ^1^PhoenixBio, Kagamiyama, 3-4-1 Kagamiya, Higashihiroshima 739-0046, Japan; ^2^Osaka City University Gradate School of Medicine, 1-4-3 Asahi-machi, Abeno-ku, Osaka 545-8585, Japan

## Abstract

Preclinical studies to predict the efficacy and safety of drugs have conventionally been conducted almost exclusively in mice and rats as rodents, despite the differences in drug metabolism between humans and rodents. Furthermore, human (*h*) viruses such as hepatitis viruses do not infect the rodent liver. A mouse bearing a liver in which the hepatocytes have been largely repopulated with *h*-hepatocytes would overcome some of these disadvantages. We have established a practical, efficient, and large-scale production system for such mice. Accumulated evidence has demonstrated that these hepatocyte-humanized mice are a useful and reliable animal model, exhibiting *h*-type responses in a series of in vivo drug processing (adsorption, distribution, metabolism, excretion) experiments and in the infection and propagation of hepatic viruses. In this review, we present the current status of studies on chimeric mice and describe their usefulness in the study of peroxisome proliferator-activated receptors.

## 1. Introduction

The human (*h*)-body consists of approximately 30 organs, each of which fulfills a specific function, autonomously yet cooperatively with other organs, to maintain life. The liver is essential to (*h*)-life, as it participates in the control of energy balance and plays central roles in the metabolism and excretion of ingested food and chemicals. Knowledge of the mechanisms underlying the functions of the *h*-liver is important for understanding the biology of the liver as well as for clinically treating liver-damaged patients and for studying drug pharmacology in humans. The ideal approach to elucidating the mechanisms responsible for liver functions would be to perform experiments using the *h*-liver *in situ*, but of course this approach is not realistic. Therefore, scientists have taken two other approaches: in vitro examination of samples isolated from the *h*-body (in vitro*/*human), and in vivo examinations using animals (in vivo/animal). Although these two approaches, separately and together, have revealed much about the mechanisms governing the functions and morphology of the *h*-liver, they are inherently limited by the complexity of the biological phenomena and the species differences in homologous mechanisms between animals and humans.

The complexity of a biological phenomenon results from the required mutual interactions of multiple different components. The specific cells that represent an organ's functions are collectively termed parenchymal cells. For example, the parenchymal cells of the liver are hepatocyte, because they perform liver-specific functions such as the synthesis and secretion of serum proteins and the synthesis of metabolism-related enzymes, including liver-specific cytochrome P450 (CYP450) proteins. However, hepatocytes by themselves are unable to fulfill liver functions and require the cooperation of nonparenchymal liver cells such as hepatic blood vessels, bile duct biliary cells, Kupffer cells, and stellate cells in the space of Disse, located between the hepatic plate and the sinusoids [1]. The portal vein is the major import route for nutrients to the liver, via the hepatic sinusoids, from the small and most of the large intestine, the spleen, and the pancreas. Nutrients and oxygen in the sinusoids and secretory proteins in the hepatocytes are exchanged through the space of Disse. Stellate cells, the major cell type producing extracellular matrix components in the liver, are located adjacent to the hepatocytes and the sinusoidal endothelial cells [2]. Hepatocytes, endothelial cells, and stellate cells represent 65, 21, and 6%, respectively, of the *h*-liver and are the main cells responsible for liver functions [1].

Interactive cooperation among different cells types is a principal way in which a multicellular entity is able to function as a living system. It is also a major source of the limitations in in vitro*/*human studies. To date, no studies have successfully reconstituted an in vitro/*h*-liver system that perfectly mimics the events that occur in the *h*-liver in vivo. This limitation has prompted a search for an in vivo/animal experimental system appropriate for providing animal data that can be extrapolated to humans. However, animal models must address the challenge of species differences in the genes and proteins associated with a biological phenomenon.

The liver processes nutrients from the gut and intestines into proteins, lipids, and carbohydrates. It also serves an endocrine function by secreting albumin (Alb), most coagulation factors, several plasma carrier proteins, and lipids into the blood. In addition, the liver synthesizes bile and secretes it into the digestive tract. The elaborate histological structure of the liver optimizes these functions [3]. Hepatocytes are well organized in an aggregated association (the hepatic epithelium) of polarized hepatocytes, creating small apical domains that line the channels between cells (canaliculi). These channels connect to the bile ducts, which drain into the intestine. The basal sides of the hepatocytes are juxtaposed to the fenestrated endothelium of the sinusoids, into which blood flows from the arterial and intestinal portal circulations before emptying into the venous circulation [4].

*h*-Hepatocytes are indispensable for an in vitro*/*human liver study. Nevertheless, the preparation of *h*-hepatocytes in sufficient numbers for experimental purposes is difficult because the source is very limited and because *h*-hepatocytes do not abundantly proliferate and grow in vitro. This led us to create a mouse (*m*) bearing a liver composed almost entirely of *h*-hepatocytes. This approach may simultaneously abolish the limitations of both in vitro*/*human and in vivo/animal studies. With this *m-*model, a small number of available *h*-hepatocytes could abundantly proliferate in the *m*-liver for use in in vitro*/*human studies. Furthermore, these mice would provide a superior new type of model animal for in vivo/animal studies, because fewer species differences would exist with respect to liver functions.

We have called this type of mouse a “liver-humanized mouse,” or simply a chimeric mouse, although the correct name should be “hepatocyte-humanized mouse.” The idea of a *h*-liver chimeric mouse was originally described by Brinster's group in 1995 [5] and was actualized by the two groups in 2001 to study *h*-hepatitis B virus (*h*-HBVs) [6] and *h*-HCV infections [7], and later, in 2004, by us to study the in vivo growth capacity of *h*-hepatocytes and the gene and protein expression of CYPs [8]. One year later, a detailed morphological study of a chimeric *m*-liver was reported by Meuleman et al. [9]. Kneteman and Mercer briefly reviewed the current chimeric mouse studies [10]. In this article, we review the studies on chimeric mice, including their short historical background, usefulness in testing *h*-type metabolism of clinically usable drugs, and potential use in examining *h*-type peroxisome proliferator-activated receptors (PPARs), especially PPAR*α*, which plays key roles in the metabolism of xenobiotics in an animal species-dependent manner. We demonstrate that *h*-hepatocytes propagated in a chimeric *m*-liver and then isolated can serve as normal *h*-hepatocytes for an in vitro*/*human model [11].

## 2. A Mouse Bearing Transplanted Homogenic and Xenogenic Hepatocytes

To study neonatal bleeding disorders, transgenic mice (Tg_Alb-uPA_) carrying a tandem array of about four Albumin promoter/enhancer-driven urokinase-type plasminogen activator (uPA) genes were created [12]. Their hepatocytes over-produce murine urokinase, and the liver becomes severely hypofibrinogenemic, which accelerates hepatocyte death. Sandgren et al*.* [13] developed a model of liver regeneration in Tg_Alb-uPA_ mice, in which a chronic stimulus for liver growth was generated due to a functional liver deficit. When a hepatocyte stochastically deleted the deleterious transgene, the hepatocytes of mice hemizygous for the transgene started to replicate and selectively expanded to regain the original size of the liver. Transgene expression in the replicating hepatocytes was abolished because of a DNA rearrangement that affected the transgene tandem array. This permitted the individuals to survive beyond birth, and the plasma uPA concentrations gradually returned to normal by 2 months of age. The transgene-deficient cells formed clonal colonies called hepatic nodules. These nodules expanded and replaced the surrounding transgene-active cells, which could not replicate because of cellular damage. Eventually, the transgene-deficient cells replaced the entire liver. This study demonstrates the usefulness of the Tg_Alb-uPA_ mouse for examining the replicative capacity of not only *m*-hepatocytes, which was successfully done by transplanting hepatocytes isolated from adult mice into the transgenic mice [14], but also hepatocytes of mammals that acquire immunotolerance as follows.

Rhim et al*.* [5] introduced the Alb-uPA transgene into immunotolerant nu/nu mice by mating Tg_Alb-uPA_ mice with Swiss athymic nude mice, generating immunotolerant Tg_Alb-uPA_ mice (Tg_Alb-uPA_/NUDE mice). Rat (*r*) liver cells were transplanted into the livers of Tg_Alb-uPA_^+/+^/NUDE mice homozygous for the transgene. The host livers that had not been transplanted with *r*-liver cells were completely pale (white). In contrast, those with *r*-liver cells had white regions, representing the area composed only of transgene-expressing host *m*-cells, and red regions, representing the area composed of transgene-deleted host *m*-cells, repopulated *r-*cells, or both. Immunohistochemical analysis with antibodies against *r*-hepatocytes confirmed that the red region was composed primarily of *r*-hepatocytes. The completely regenerated transgenic *m*-livers resemble normal *m*-livers in color, shape, and size. Southern blot analysis demonstrated that up to 56% of the DNA was of rat origin, which agreed well with the parenchymal cell occupancy rate in the liver. These findings strongly support the idea that the host liver was chimeric, with *r*-parenchyma and *m*-nonparenchymal cells, which included vessels, bile ducts, and associated connective tissues. The ratio of the liver weight to the body weight was 6.8%, which was similar to that of the non-transgenic control mice (5.8%), indicating that the rat-mouse (*r/m*) chimeric livers were able to normally terminate growth. The successful generation of a healthy mouse with a chimeric liver indicates that *r*-parenchymal and *m*-nonparenchymal cells were able to communicate with each other to reconstitute a functional liver, despite the species difference.

Hepatocytes initiate and terminate proliferation under the influence of nonparenchymal cells [1]. Thus, the normal progression and termination of *r/m*-chimeric liver regeneration implies that* r*-hepatocytes produce surface proteins that interact correctly with soluble *m*-factors, *m*-extracellular matrix, and *m*-surface proteins on *m*-nonparenchymal cells. The successful replacement of Tg_Alb-uPA_^+/+^/NUDE*m-*livers with *r*-hepatocytes raised the exciting possibility that *m*-livers could also be reconstituted with *h*-hepatocytes [5].

## 3. Repopulation of *h*-Hepatocytes in *m*-Liver

**Figure 1:**
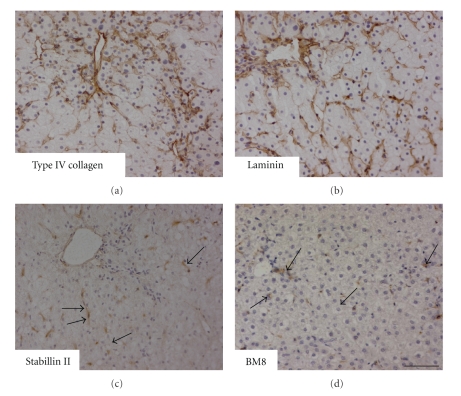
The histological harmonization of *h*-hepatocytes with* m*-nonparenchymal cells. uPA/SCID mice were transplanted with *h*-hepatocytes and allowed to grow until the repopulation of the liver was complete. Then, liver sections were prepared from the *h*-hepatocyte-chimeric mice. Sections were immunostained with *m*-specific antibodies for type IV collagen (a); laminin (b); stabillin (c), a marker of liver endothelial cells (a gift from Dr. A. Miyajima, Tokyo University); and BM8 (d), a marker of Kupffer cells. The immunosignals are brown. The arrows in (c) and (d) point to typical immunopositive cells. Bar, 100 *μ*m.

**Figure 2:**
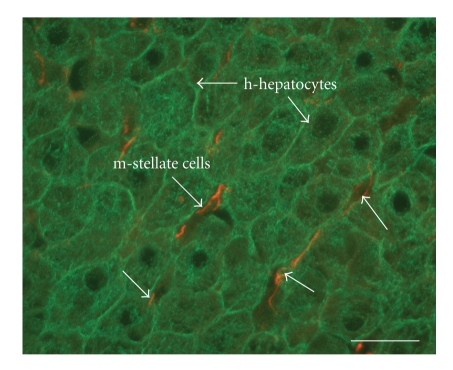
Close natural apposition of *m*-stellate cells and *h*-hepatocytes in a chimeric liver. A serial section shown in Figure 1 was doubly stained with *h*-CK8/18 (green) for *h*-hepatocytes and *m*-desmin (orange) for *m*-stellate cells. The *h*-hepatocytes are well organized and closely apposed to *m*-stellate cells in Disse's space. Arrows indicate representative *h*-hepatocytes (green) and *m*-stellate cells (orange). Bar, 10 *μ*m.

In two previous studies to generate a mouse with a *h*-hepatocyte-mouse (*h/m*) chimeric liver, Rug-2-knockout mice [6] and severe combined immunodeficient (SCID) mice [7] were used as immunodeficient mating partners for uPA transgenic mice. We mated SCID mice (mice_SCID_) with Tg_Alb-uPA_^+/+^ mice to yield liver-injured SCID mice (mice_Alb-uPA/SCID_) [8]. Normal *h*-hepatocytes, ~10^6^ viable cells per mouse, were transplanted into the livers of these mice at 20–30 days after birth. The *h*-hepatocytes engrafted the liver at rates as high as 96% and progressively repopulated it. The repopulation after *h*-hepatocyte transplantation was easily monitored by the increase in the *h*-Alb concentration in the host blood, and the expansion of *h*-hepatocyte colonies was visualized by immunohistological staining of liver sections with *h*-specific anti-cytokeratin (CK) 8/18 antibodies. The ratio of the number of engrafted *h*-hepatocytes to total hepatocytes (*m*- and *h*-hepatocytes) in the host liver, which is the replacement index (RI), was determined by calculating the ratio of the area occupied by hCK8/18-positive hepatocytes to the entire area examined in immunohistochemical sections of seven lobes. It was demonstrated that sustained engraftment of *h*-hepatocytes occurs in homozygous Alb-uPA transgenic (Tg_Alb-uPA_^+/+^) mice, but not in hemizygous transgenic (Tg_Alb-uPA_^+/−^) mice. The *h*-hepatocytes started to proliferate around 7 days after transplantation. Their colonies gradually became larger and were almost confluent at around 70 days, when the RI was as high as 96%. Immunohistological staining of liver sections for type IV collagen, laminin, stabilin (a liver endothelial cell marker), BM8 (a Kupffer cell marker), and desmin (a hepatic stellate cell marker) demonstrated the chimeric nature of the liver (Figure 1). The interactions between hepatocytes and stellate cells are critical for physiological and pathological conditions of the liver [15]. Close and seemingly normal associations of *h*-hepatocytes with *m*-stellate cells were immunohistologically visualized by staining with specific antibodies against *h*-CK8/18 (*h*-hepatocytes) and *m*-desmin (*m*-stellate cells) (Figure 2). These results clearly show that the chimeric *m*-livers with a high RI consisted of parenchymal cells (mostly *h*-cells with a low percentage of *m*-cells), *m*-nonparenchymal cells, and *m*-ECMs, in agreement with a previous study [9]. There was good correlation between the RI and the mRNA expression levels of housekeeping genes such as *h*-Alb and *h*-transferrin, supporting the notion that transplanted *h*-hepatocytes are functional [16]. In our experience, mice with >6 mg/mL *h*-Alb in the blood had an RI >70%. Our histological studies illustrated that the *h*-hepatocytes were well organized and surrounded by *m*-nonparenchymal cells, and they reconstituted the normal tissues specific to a normal functional liver (described in Section 1), despite the large species difference between humans and mice.

We chose robustly growing young mice as hosts. These mice were able to not only survive but also grow, although relatively slowly, and increase their body weight by >50% of their original weight, during the replacement of host *m*-hepatocytes with* h*-counterparts. These simple animal experiments made us realize that *m*-cells and *h*-hepatocytes were able to mutually communicate to maintain life: *m*-cells supported the proliferation of *h*-hepatocytes, and *h*-hepatocytes supported the growth of the young mouse. The host liver of a mouse_Alb-uPA/SCID_ is congenitally damaged owing to uPA overproduction, low blood levels of Alb, and significantly high levels of alanine aminotransferase (ALT). Repopulation of the *h*-hepatocytes in the liver increased the blood Alb concentration and decreased the ALT level, indicating that *h*-hepatocytes contributed to the improvement of *m*-liver function [8]. Based on these considerations and findings, we expect that a *m*-liver made of *h*-hepatocytes would function as an apparently normal liver, metabolizing and detoxifying endogenous and exogenous biomolecules.

## 4. Expression Profiles of *h*-Cytchrome P450s in Relation to Phase 1 Metabolic Enzymes

Biochemical treatment of foreign substances (xenobiotics) that have been absorbed into the body is one of the major tasks of the liver. In hepatocytes, xenobiotics are processed to more stable and hydrophilic derivatives by groups of enzymes, collectively called xenobiotic-metabolizing enzymes (XMEs), via two phases: phase 1, which is accomplished by oxidative enzyme, and phase 2, performed by conjugating enzymes [17]. Ingested drugs, toxicants, and chemical carcinogens are metabolized in phase I by CYP and the flavin-containing monooxygenase superfamily. Notably, CYP is the key enzyme in the elimination of clinical drugs.

Humans and rodents respond differently to xenobiotics, and this is explained in part by species differences in CYP subfamilies. These species differences raise serious issues in research for clinically usable medicines, because the results of xenobiotic metabolism studies with mice and rats, which are the most commonly used experimental models for pharmacological and toxicological studies, cannot be extrapolated to humans. Thus, information about the expression of CYP families and subfamilies should be valuable from two viewpoints. First, the expression in *h/m*-chimeric mice of a CYP subtype that is found in *h*-hepatocytes, but not in mice, would be a good indication that the *h*-hepatocytes are biochemically functional in the *m*-iver. Second, the *h-*CYP-expressing chimeric mouse is a useful experimental model for studying *h*-type metabolic responses to xenobiotics, including clinically valuable drugs. Of note, CYP3A4 is the most abundantly expressed CYP in *h*-liver and metabolizes >60% of all therapeutic drugs; collectively, CYP2D6 and CYP3A4 metabolize >70% of the drugs on the market [17].

Species differences in the CYP2C subfamily are well known and have been characterized intensively [18, 19]. The *h*-liver contains four CYP2C isoforms, CYP2C8, CYP2C9, CYP2C18, and CYP2C19, all of which are absent from mice and rats. Western blot analyses using *h*-specific antibodies against CYP2C9 revealed positive signals with hepatocytic microsomal fractions from *h/m*-chimeric mice with an RI >34% and from the donor, but not with hepatocytic microsomal fractions from chimeric mice with an RI <28% or from mice that had not been transplanted with *h*-hepatocytes. CYP2C9 catalyzes the 4′-hydroxylation of diclofenac, and the microsomal fractions from the chimeric mice showed diclofenac 4′-hydroxylation activity that depended on the RI of the mouse, strongly suggesting that the *h*-hepatocytes in chimeric livers retain *h*-type pharmacological activity toward drugs. One of the clearest and best-defined examples of a difference in a CYP between mice and humans is CYP2D6 [20, 21], which is involved in the metabolism of a large number of clinically used drugs [22, 23]. In humans, CYP2D6 is the only active member of the CYP2D subfamily, whereas rats and mice do not express a protein with the enzyme activity of *h*-CYP2D6, although they do have at least five other CYP2D genes [20, 24]. The enzymatic activity of *h*-CYP2D6 in the chimeric mouse was demonstrated by orally administering debrisoquin, a *h*-CYP2D6 substrate, to the mice and subsequently detecting 4′-hydroxydebrisoquin, a major debrisoquin metabolite produced by *h*-CYP2D6, in the blood of the mice. Pretreatment of the mice with quinidine, a typical *h*-CYP2D6 inhibitor, decreased the level of the metabolite. Thus, a CYP enzymatic activity in the chimeric mice was specifically induced by a CYP2D6-metabolized drug and specifically suppressed by a CYP2D6 inhibitor [25].

Among the known CYP families, four families (CYP1–4) play primary roles in XMEs. We compared the mRNA and protein expression profiles of six *h*-CYPs, CYP1A1, 1A2, 2C9, 2C19, 2D6, and 3A4, in the chimeric *m*-liver with those in the donor liver [8]. Total RNA was prepared from the livers of chimeric mice with different RIs and of donors, and the mRNA for the six *h*-CYPs was amplified in a quantitative reverse-transcriptase polymerase chain reaction (qRT-PCR). All six mRNAs were amplified to detectable levels, which were higher in mice with higher RI values. Thus, the *h*-hepatocytes in the chimeric mice appeared to express the six *h*-CYP genes in a manner similar to their expression in the *h*-body.

We then asked whether these normally expressed *h*-CYPs in the *h/m*-chimeric liver were inducible in a drug-specific manner. The *h*-CYP3A4 and *h*-CYP1A subfamilies specifically respond to rifampicin and 3-methylcholanthrene (3-MC), respectively [26]. Chimeric mice with *h*-hepatocytes were injected intraperitoneally with rifampicin or 3-MC, once per day for 4 days. The mRNA levels of the six *h*-CYPs in the liver tissues were measured 24 h after the last injection. Rifampicin treatment enhanced the expression of *h*-CYP3A4 by 5.8-fold, but did not affect the levels of the other five *h*-CYPs. The administration of 3-MC enhanced CYP1A1 and CYP1A2 mRNA levels by 10.0-fold and 6.4-fold, respectively, but had no effect on the other four CYPs. Neither rifampicin nor 3-MC induced the expression of any of the six *h*-CYPs in mice_Alb-uPA/SCID_ that had not been transplanted with *h*-hepatocytes. Rifabutin, an analogue of rifampicin, also specifically induced *h*-CYP3A, but not the host murine Cyp3a, in the chimeric *m*-liver [27]. The degree of CYP3A4 induction in the chimeric mouse has practical applications in drug testing, because many drugs are CYP3A4 substrates and the induction of CYP3A4 decreases the pharmacological potency of these drugs [17].

Rifampicin is a ligand for the pregnane X receptor (PXR), which forms a heterodimer with retinoid X receptor a (RXRa). Rifampicin/PXR/RXRa subsequently binds to a xenobiotic response element (XRE) composed of the direct repeat of alpha and beta half-sites separated by four nucleotides on the CYP3A4 gene, thereby upregulating its expression in phase 1 [28]. Rifampicin is a potent activator of human and rabbit PXR, but has little activity in the rat and mouse [29]. Thus, that the liver data of *h/m*-chimeric mice faithfully reflect those in humans. The binding of 3-MC to the aryl hydrocarbon receptor (AHR) forms a AHR/3-MC complex, which upregulates CYP1A1, CYP1A2, and CYP1B1 expression by binding, together with the AHR nuclear translocator (ARNT), to the XREs of these genes [30]. Our studies suggest that these known ligand-activated receptor signaling pathways activated by rifampicin and 3-MC are functional in the *h/m*-chimeric *m*-liver. Thus, we propose that the hepatocyte-humanized mouse will be a useful animal model in studies of *h*-type signaling pathways that regulate gene expression induced by xenobiotics.

## 5. Humanization of Phase II Conjugation Pathway of a Drug in *h*/*m*-Chimeric Mice

It is estimated that phase II conjugation accounts for approximately >30% of drug clearance [31], especially of compounds with polar groups. The major hepatic phase II enzymes in humans are UDP-glucuronosyltransferase (UGT), which is responsible for glucuronidation; sulfotransferase (SULT), for sulfation; *N*-acetyltransferase (NAT), for acetylation; and glutathione *S*-transferase (GST), for glutathione conjugation. We examined the mRNA and protein expression and the enzyme activity of the *h*-forms of these enzymes in chimeric mice with livers having RI values ranging from 0 to 90% [32]. The chimeric livers expressed *h*-UGT, *h*-SULT, *h*-NAT, and *h*-GST mRNA and the UGT2B7, SULT1E1, SULT2A1, and GSTA1 proteins at levels that correlated with their RI values. Activities of related enzymes such as morphine 6-glucuronosyltransferase and estrone 3-sulfotransferase were also detected in an RI-dependent manner. The protein content and enzyme activities of phase II-associated enzymes in chimeric *m*-livers with an RI of approximately 90% were similar to those in the donor liver. In a separate study, we systematically compared the mRNA expression profiles for 26 phase II *h*-enzymes, including GST, SUL, NAT, and UGT members, between livers of chimeric mice with RIs of 71–89% and donor livers [16]. All of the tested enzyme genes were detected. For 65% of the tested genes, the expression levels in the chimeric livers were 30 to 55% of the levels in the donor livers; although lower, these values are comparable to the RI values. These results indicate that the hepatic phase II biotransformation of a drug is appreciably humanized in the *h/m*-chimeric mouse.

There are groups of drugs in clinical use that bind to PXRs or constitutive androstane receptors (CARs). The ligand-activated PXRs and CARs are involved in the regulation of some phase II XME genes such as SULT1A, UGT1A, and GST [33–35]. Thus, it is likely that these *h*-type ligand-activated transcriptional regulators are functional in *h/m*-chimeric *m*-livers, suggesting that these chimeric mice will contribute to studies on the regulation of gene and protein expression of these transcription factors in relation to xenobiotic metabolism.

## 6. Drug Transport through the Chimeric *m*-Hepatocyte Membrane

Drug transport in the liver is largely performed by two systems: extrahepatic-to-hepatic transport using transporters such as organic cation transporter 1 (OCT1), organic anion transporting polypeptide (OATP) 1B1, and OATP1B3; and hepatic-to-bile duct transport using adenosine 5′-triphosphate-binding cassette (ABC) proteins, including P-glycoprotein, bile salt export pump (BSEP/ABCB11), breast cancer resistance protein (BCRP/ABCG2), and multidrug resistance-associated protein 2 (MRP2) [36]. The former transporters are located on the sinusoidal membrane and are responsible for the uptake of drugs into hepatocytes; the latter are on the canalicular membrane and are responsible for biliary excretion of the metabolites. The *h*-genes of these transporting systems were preferentially expressed compared with the *m*-counterpart genes in chimeric mice with RIs >60% [36]. Cefmetazole (CMZ), a cephalosporin antibiotic, is excreted without any chemical modification, through urinary and biliary pathways. The urinary pathway is dominant in humans [37], whereas rats [38] and mice [36] use the biliary pathway. Before receiving *h*-hepatocytes, the host mice excreted CMZ primarily through the biliary pathway, but the urinary pathway was dominant in chimeric mice with RIs >60% [36].

The *h*-ABCB4 transporters have been characterized in relation to fibrate-metabolism [39]. In addition, we examined the expression levels of 21 *h*-transporter genes, including members of the ABC, solute carrier (SLC), and OATP families, in the livers of chimeric mice with RIs ranging from 71 to 89%, with respect to the levels in donor livers [16]. For 62% of the tested genes, the expression ratios in the chimeric livers were 0.35 to 0.75. From these limited data, it appears that most of the *h*-type transporter genes were expressed in the chimeric *m*-liver.

## 7. Infectivity of Chimeric Mice with *h*-Hepatitis Viruses

*h*-Liver diseases caused by HBV and HCV, especially HCV, are targets for the discovery of efficient antivirus drugs, worldwide [40]. However, the development of effective therapeutics has been hampered by the lack of useful in vitro and in vivo models of viral replication. For example, cultured *h*-hepatocytes are not appropriate as recipient cells for viral propagation, and rodents are not useful animal models because of the strict species specificity of viral infection [41]. Viral infectivity and propagative potential in the *h/m*-chimeric mouse would be persuasive evidence for concluding that it was actually “humanized.” A research group led by Kneteman first challenged chimeric mice with an inoculation of HCV-infected *h*-serum, which produced a virus-infected model mouse [7]. Owing to their substantial advantage in both magnitude and duration of *h*-hepatocyte engraftment, homozygous animals were superior to their hemizygous counterparts in this regard. Initial increases in total viral load were up to 1950-fold, with replication confirmed by the detection of negative-strand viral RNA in transplanted livers. HCV viral proteins were localized to *h*-hepatocyte nodules, and infection was serially passed through three generations of mice, confirming both synthesis and release of infectious viral particles. Using *h*-hepatocyte-chimeric Rug-2-knockout mice as test animals, Dandri et al. was the first to succeed in producing in vivo HBV infection [6].

We studied HBV infectivity in the chimeric mice [42]. After mice were inoculated with *h*-serum containing HBV, a high level of viremia occurred in mice for up to 22 weeks. Passage experiments showed that the serum of these mice contained infectious HBV. As shown previously for HCV, the level of HBV viremia tended to be high in mice with a continuously high RI. Furthermore, lamivudine, an anti-HBV drug, effectively reduced the level of viremia in the infected mice. Thus, the chimeric mouse may be an ideal model in which we can develop and evaluate anti-*h*-hepatitis virus drugs.

## 8. The *h*/*m*-Chimeric Mouse as an Animal Model for the Study of *h*-Type Peroxisome Proliferator-Activated Receptors

### 8.1. Drug Metabolism under The Control of Ligand-Activated Receptors

Biochemical systems in the liver manage not only endogenous (homobiotic), but also xenobiotic molecules. These molecules are first recognized by specific protein receptors on the hepatocyte surface. In general, the binding of a ligand to its receptor generates a signal that ultimately changes gene expression, producing a cellular response. Hepatocytes possess four types of receptors [16], all of which are ligand-activated transcriptional regulators: CAR; PXR [also called steroid X receptor (SXR)]; peroxisome proliferator-activated receptor (PPAR); and aryl hydrocarbon receptor (AHR). The first three belong to the nuclear receptor (NR) superfamily, which consists of seven subfamilies, 1 to 6 and 0 [43]. AHR is a member of the Per-AhR/Arnt-Sim homology sequence (PAS)/basic helix-loop-helix (HLH) superfamily, which also represents the period regulator of circadian rhythm (PER), Ah receptor nuclear translocator (ARNT), and single-minded regulator of midline cell differentiation.

Historically, the roles of PPARs have been studied using liver. They belong to NR subfamily 1, along with thyroid hormone receptor, retinoic acid receptor (RAR), and vitamin D receptor (VDR). As transcription factors, these receptors share a similar process. They are activated by ligand binding; form heterodimers, usually with the retinoid X receptor (RXR); translocate to the nucleus; bind to a *cis*-acting XRE consisting of a direct repeat of two hexanucleotides, separated by one or two nucleotides, in the promoter region of the target gene; and enhance target gene expression [17]. Generally, in the absence of ligand, subfamily 1 NR heterodimers are bound to co-repressor proteins and repress transcription when bound to the *cis*-acting element [44]. Upon ligand binding, the receptor dissociates from the co-repressors and associates with coactivator proteins, which enables the NRs to promote gene expression.

Three PPAR subtypes are currently known [45]: PPAR*α* (or NR1C1), PPAR*β*/*δ* (NR1C2), and PPAR*γ* (NR1C3). When continuously exposed to certain xenobiotics such as hypolipidemic drugs, plasticizers, and herbicides, which have little apparent structural relationship, rats and mice may show hepatic peroxisome proliferation (increase in volume and number) leading to hepatic tumors; this suggests a correlation between the stimulation of genes for fatty acid *β*- and *ω*-oxidation enzymes and the hepatic neoplastic process [46, 47]. Reddy and Rao [48] proposed that specific soluble binding sites for these drugs, collectively termed peroxisome proliferators (PPs), were present in liver and kidney cell extracts [49, 50]. The PPAR gene was first cloned as a member of the steroid hormone receptor superfamily from a *m*-hepatic cDNA library [51]. This gene corresponds to PPAR*α*, according to the current nomenclature. Two years later, three closely related members of the PPAR family (xPPAR*α*, *β*, and *γ*) were isolated from a *Xenopus* ovary cDNA library and were shown to activate the promoter of the acyl coenzyme A oxidase (ACO) gene, which encodes the key peroxisomal fatty acid *β*-oxidation enzyme [52]. xPPAR*α* is homologous to Issemann's PPAR*α* [51], and xPPAR*γ* is currently placed in the PPAR*γ* subfamily, together with other homologous members found in mammals. Mammalian PPAR*δ* was in a new PPAR group because of a difference in amino acid sequence compared with xPPAR*β*; however, it is presently considered to be a PPAR*β* and is designated as PPAR*β*/*δ* [45]. Of the three PPARs, PPAR*α* is the most critical in the present review, because it is expressed at high levels in the liver, activates fatty acid catabolism, stimulates gluconeogenesis and ketone body synthesis, and participates in the control of lipoprotein assembly [45].

### 8.2. Species Differences in PPAR*α*-Associated Signaling

The PPAR*α* isotype has prime importance for studies with animal models to predict the effects of hepatic PPs in humans, because PPAR*α* agonists induce seemingly quite different actions in rodents and humans [53]. Originally, as the name indicates, PPARs were studied because of their ability to bind PPs and consequently induce PP-metabolizing enzymes. In rats and mice, but not in humans, PPs such as hypolipidemic drugs, industrial plasticizers, and herbicides are non-genotoxic carcinogens that cause liver tumors [54]. In humans, these drugs function to maintain lipid homeostasis and do not induce peroxisome proliferation. Thus, the toxicity and carcinogenicity of PPs are highly species specific [55]. The species differences may be attributable to lower PPAR mRNA expression levels in *h*-hepatocytes compared with rodent cells [56, 57]. Alternatively, or additionally, species differences may be the result of different sensitivities of the genes associated with the peroxisome proliferation response to low levels of PPs, owing to structural differences in PPAR*α* [54]. There are both similarities and differences in responses to xenobiotics among not only different species (interspecies) but also individuals of the same species (intraspecies). Interspecific PPAR*α* diversity between rodents and humans is well known and has been studied with respect to drug metabolism. NR subfamily 1 members have at least two functions in mammals. One is to regulate peroxisome proliferation through binding to PPAR response elements (PPREs) in the promoters of genes such as ACO [58, 59], bifunctional dehydrogenase/hydratase (BFE) [60], and microsomal CYP4A1 [61]. The other is to modulate the serum cholesterol level by targeting genes such as the lipoprotein lipase gene [62] and the apolipoprotein regulating genes AI, AII, and CII [63]. The former mechanism appears to function in rodents, but not in humans, and is responsible for the induction of peroxisome proliferation and hepatocarcinogenesis, whereas the latter mechanism controls basic lipid metabolism in both rodents and humans [56]. This species difference in xenobiotic receptor/ligand signaling may be attributable to differences in the expression level of a receptor, or to differences in receptor/ligand binding affinity, and causes difficulty in determining responses in humans based on rodent data [56].

### 8.3. PPAR*α* Gene-Humanized Mice

One approach to overcoming species differences is to generate “humanized” transgenic mice (gene-humanized mice), in which a *h*-gene of interest is introduced into the *m*-genome [17]. A PPAR*α* gene-humanized *m*-line that expresses the *h*-PPAR*α* gene [64] under the control of the tetracycline responsive regulatory system in the liver of murine PPAR*α* gene-null mice [65] has been created. These mice functionally responded to the expected ligands as wild-type mice, but did not exhibit the hepatocellular proliferation, including increases in peroxisomes, seen in wild-type mice. Thus, this approach may help overcome species differences and provide animal models suitable for studying *h*-responses regulated by genes of interest.

### 8.4. PPAR Signaling in Chimeric Mice

Considering the prominent roles and the species divergence of PPARs in the response to xenobiotics, it is important to study *h*-PPAR-related responses of the *h/m*-chimeric mouse. We examined the effects of fibrates (antihyperlipidemic drugs and PPAR agonists) [63, 66] in the chimeric mice. Given the central role of the liver in PPAR-regulated lipid metabolism and the use of fibrate compounds in a variety of clinical drugs, the responses of *h/m*-chimeric mice to fibrates and PPARs may have important practical implications.

Hepatocytes secrete biliary phospholipids, composed largely of phosphatidylcholine (PC), through multidrug-resistance 2 P-glycoprotein (MDR3, or ABCB4) embedded in the canalicular membrane. MDR3 was shown to translocate PC in a study using mdr2 gene (a murine homolog of *h*-MDR3) knockout mice. These mice completely lack phospholipids in their bile [67], but the bile PC is fully recovered with the overexpression of *h*-MDR3 [68]. The expression level of the *h*-MDR3 gene affects the development of hepatobiliary diseases [69].

Fibrates upregulate mdr2 gene expression [70], which is associated with an increase in biliary phospholipid secretion [71]. Benzafibrate (BF), a second-generation fibrate analog, was clinically shown to reduce elevated serum biliary enzyme levels in patients with chronic cholestatic liver disease [72]. It was shown to bind to PPAR*β*/*δ* and *α*, with a higher affinity for the former, and was thus said to be a bona fide PPAR ligand [73]. Other researchers created a coactivator-dependent receptor-ligand in vitro interaction assay and demonstrated that BF was a ligand for PPAR*α*, *β*/*δ*, and *γ* [74]. The same researchers also showed drug-induced activation of PPAR*α*/RXR*α*, PPAR*β*/*δ*/RXR*α*, and PPAR*γ*/RXR*α* [74].

BF induces an increase in ABCB4 (MDR3), and its redistribution in the cell membrane. This induction was associated with an enhanced capacity of *h*-hepatocytes to direct PC into bile canaliculi [75]. Furthermore, ABCB4 redistribution was attenuated when PPAR*α* expression was suppressed by small interfering RNA or morpholino antisense oligonucleotides in cultured HepG2 cells (hepatoblastoma cells) [75], strongly suggesting the necessity for PPAR*α* in the BF-induced activation of PC secretion in *h*-hepatocytes.

We tested the ability of the *h/m*-chimeric *m*-liver to exhibit *h*-type PPAR-dependent responses by administering BF to the chimeric mice. Mice with RIs of 60–80% were fed a standard laboratory chow containing 0.3% (wt/wt) BF for 7 days, and their livers were analyzed for MDR3 mRNA and protein expression [39]. The mRNA level in the BF-treated mice was approximately 2-fold the level in non-treated control mice. The protein level was approximately 3.5-fold that in the controls. The fibrate induced a robust redistribution (exocytosis and insertion) of MDR3 proteins into the bile canaliculi.

Although studies on the expression and function of PPARs in the *h/m*-chimeric *m*-liver are limited, we conclude, based on the studies described above, that the chimeric *m*-liver exhibits the phenotypes of PPAR-regulated physiological and pathological processes, including responses to xenobiotics, that are normally present in the *h*-liver in vivo.

## 9. Summary and Prospective

After administration, a xenobiotic is generally and largely absorbed by the liver, intracellularly distributed, metabolized, and secreted through the bile or urinary ducts. These steps, collectively termed absorption, distribution, metabolism, and excretion (ADME), are interdependent, and drug pharmacokinetics are determined by the parameters resulting from these interactive processes. There are marked species differences in the many genes and proteins associated with ADME of a xenobiotic. The differences between humans and rodents dictate that pharmacokinetic data determined in rodents must be very cautiously, deliberately, and correctly extrapolated to humans in order to ensure that the drug will be safe and effective in patients. Until recently, *h*-hepatocyte-chimeric mice have been studied primarily in relation to CYP-associated metabolism, representing the M of ADME, and HCV/HBV infection. These studies have shown that the chimeric mice are significantly and appreciably humanized, providing a reliable and promising animal model for predicting drug metabolism and efficacy in humans. Although data have also been accumulated for the A, D, and E steps of ADME, more work is required before reaching an appropriate conclusion concerning the humanization of a chimeric mouse with respect to these processes. Nevertheless, currently available data appear to demonstrate that these processes are also well humanized.

Based on our studies and experiences to date, the *h*-hepatocyte-chimeric mice exhibit *h*-type liver responses at the gene and protein levels. These mice can mimic the steady-state expression in the *h*-liver in the absence of exogenous stimuli and exhibit the expected *h*-type responses upon stimulation. However, we must consider the limitations of chimeric mice. Current chimeric mice carry hepatocytes only of human origin, but all other cells are of *m*-origin. To perform liver functions, parenchymal cells require nonparenchymal cells, which are of mouse, and not of human, origin in the chimeric mice. Some interactions between *h*-hepatocytes and *m*-nonparenchymal cells may proceed as normal homogeneous interactions, and some may not.

In addition, endocrinological regulation is crucial for hepatocytes to achieve normal metabolic homeostasis and to return to normal conditions after endogenous or exogenous factors have caused metabolic parameters to extend beyond the normal range. Chimeric livers are under the influence of the *m*-endocrinological system, and some *m*-hormones such as growth hormone (GH) are not able to act on *h*-cells, because a hormone-receptor complex does not form between *m*-GH and *h*-hepatocyte receptors [76]. In support of this notion, *h*-hepatocytes administered with *h*-GH showed enhanced expression of liver growth-associated *h*-genes, including IGF-1, STAT-3, Cdc 25A, and cyclinD1, and repopulated the host liver at a rate approximately 6-fold that in the control. Despite these possible limitations, we consider the chimeric mouse to be the best animal model to date for *h*-liver function studies, because the chimeric mice with high RI values not only expressed *h*-liver proteins but also mimicked *h*-liver functions. Five years ago, we started mass production of homogenous populations of the hepatocyte-humanized mice with high RIs to facilitate research activities in the academic and industrial communities, including examinations of *h*-type metabolism of new drugs for *h*-use, the study of *h*-HCV infection mechanism and propagation, and the development of new anti-HCV-drugs. However, we are still in the initial stages of characterizing various aspects of the chimeric mice. Further study will systematically reveal the advantages and limits of this newly developed hepatocyte-humanized mouse.
